# Graphene oxide-enhanced sol-gel transition sensitivity and drug release performance of an amphiphilic copolymer-based nanocomposite

**DOI:** 10.1038/srep31815

**Published:** 2016-08-19

**Authors:** Huawen Hu, Xiaowen Wang, Ka I Lee, Kaikai Ma, Hong Hu, John H. Xin

**Affiliations:** 1 Foshan University, Guangdong, 528000, China; 2 The Hong Kong Polytechnic University, Hong Kong SAR, 999077, China

## Abstract

We report the fabrication of a highly sensitive amphiphilic copolymer-based nanocomposite incorporating with graphene oxide (GO), which exhibited a low-intensity UV light-triggered sol-gel transition. Non-cytotoxicity was observed for the composite gels after the GO incorporation. Of particular interest were the microchannels that were formed spontaneously within the GO-incorporated UV-gel, which expedited sustained drug release. Therefore, the present highly UV-sensitive, non-cytotoxic amphiphilic copolymer-based composites is expected to provide enhanced photothermal therapy and chemotherapy by means of GO’s unique photothermal properties, as well as through efficient passive targeting resulting from the sol-gel transition characteristic of the copolymer-based system with improved sensitivity, which thus promises the enhanced treatment of patients with cancer and other diseases.

Using thermal treatment, many kinds of amphiphilic block polymers have been demonstrated to exhibit sol-gel transition behaviors, as driven by the aggregation of self-assembled micelles^1^,^2^,^3^,^4^. The gel thus-formed is also known as thermogels, most of which feature the human body temperature-induced sol-gel transition^5^,^6^,^7^ that is highly useful in biomedical fields, *e.g.* to treat cancer^8^ and to deliver cell growth factor^9^ for human healthcare. Through simple intravenous administration, a starting sol loaded with drugs can be transferred quickly to a gel on the affected part of a human body for *in vivo* sustained drug release-based chemotherapy; this has been widely exploited, leading to a dilemma. It is therefore highly desirable to develop new mechanisms, so as to fabricate better materials for providing enhanced therapies for the treatment of patients with cancer and other diseases.

This article for the first time explores the feasibility of using graphene oxide (GO), a popular carbon material in various scientific disciplines including biomedicine^7^,^10^,^11^,^12^,^13^,^14^, etc. to make an amphiphilic copolymer, poly (ethylene glycol) methyl ether (mPEG)-poly(ε-caprolactone) (PCL)-mPEG, highly UV light-sensitive, which leads to the generation of a new smart nanocomposite material promising potential biomedical applications. In fact, many reports have discussed that GO and its reduced counterparts, namely reduced graphene oxide (rGO), can be used as a near-infrared (NIR) light absorber for non-invasive photothermal therapy such as *in vivo* tumor ablation, by virtue of the remarkable photothermal properties of GO or rGO^15^,^16^,^17^,^18^. However, GO, rGO or their modified derivatives (such as PEG-functionalized graphene) are, normally, in a sol state both before and after intravenous administration, which most likely results in a low administration efficacy and even could generate a side effect to normal cells, especially considering that some of the materials showed only limited passive tumor uptake in the absence of a targeting ligand^19^. Herein, we present a new concept that is believed to achieve efficient passive targeting through an enhanced sol-gel transition based on amphiphilic copolymer and graphene chemistries, *e.g.* a starting sol can quickly turn into an immobilized gel after injected to the targeted tumor location. By virtue of GO’s large specific surface area, and superior optical absorption and photothermal conversion^15^, the concept is expected to achieve enhanced photothermal therapy and chemotherapy, *e.g.* increasing the drug loading capacity and enhancing the heat generation. After encapsulated in polymer matrices, GO has also been well demonstrated to possess low toxicity and high biocompatibility^10^.

Of particular interest is that the GO incorporation could impart an enhanced sol-gel transition sensitivity to the amphiphilic copolymer mPEG-PCL-mPEG, in this case a low-intensity UV light sensitivity (the gel thus-formed is labeled as UV-gel, and those with the lower and higher GO contents are further designated as 0.2GO and 2.0GO respectively), while no sol-gel transition could be observed for our pure copopymer system under the same UV conditions. Many studies have focused on using NIR light for photothermal therapies in that an appreciable thermal effect of NIR light can be generated on most objects (especially graphene materials showing strong NIR light absorption), as a result of wave resonance^10^,^20^,^21^. By contrast, UV light has a limited thermal effect despite its unique chemical effects such as fluorescence and disinfection. Therefore, it is a significant finding that GO is capable of endowing the amphiphilic copolymer with a sol-gel transition under UV light (365 nm) at a low intensity (0.8 mW/cm^2^). This also implies that GO affords a significantly high sensitivity to the amphiphilic copolymer in terms of obtaining a sol-gel transition.

It has been reported that nano- or micro-channel based drug delivery technologies presents an unprecedented opportunity to control drug release kinetics in delivery devices^22^. Microchannels have also been created intentionally and used for drug delivery^22^,^23^,^24^. Here, microchannels were formed spontaneously within the UV-gel as induced by a directing effect of GO, which could confer a reduced diffusion barrier and hence an increased drug release rate to the UV-gel; this is an advantage over the traditional drug carrier (in most case rGO) with biocompatible linker polymers, *e.g.* PEG^25^ and glucose^26^, that are diffusion barriers^27^. As a consequence, the work presented here opens up an avenue to fabricate newly structured block copolymer-based composite materials with microchannels using the graphene chemistry. This study will also shed light on the fabrication of copolymer-based nanocomposites incorporating with light-sensitive carbon nanomaterials or metal nanoparticles for enhanced photothermal therapy and chemotherapy.

## Results

**Figure 1:**
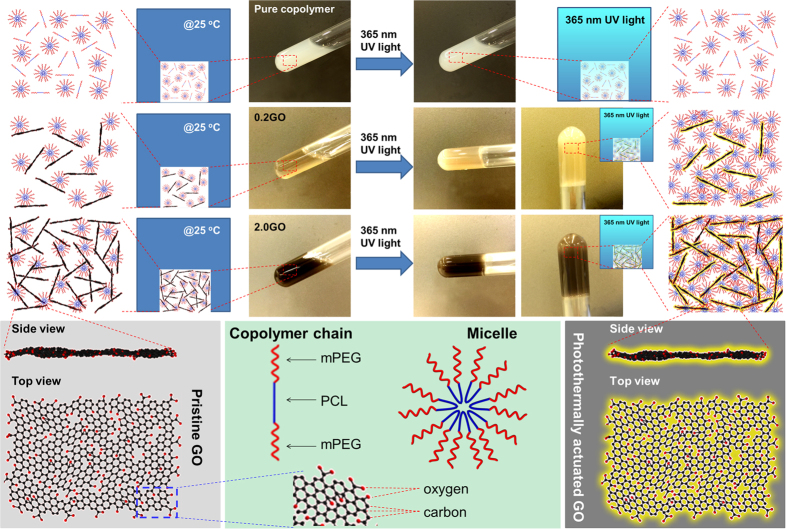
Schematic illustration of the UV light-driven sol-gel transition of the copolymer mPEG-PCL-mPEG based composite filled with different contents of GO being used as the photosensitizer. UV light source: 365 nm, 0.8 mW/cm^2^.

Figure 1 illustrates the fabrication of UV-sensitive amphiphilic copolymer-based composites with GO as a UV light sensitizer. From the photoimages and schematic diagrams, a clear sol-gel transition can be noted for the composite systems after the present 365 nm UV light irradiation at a low intensity (0.8 mW/cm^2^) for only 10 min, whereas such a UV-driven sol-gel transition cannot take place for the pure copolymer system due to a limited thermal effect of the UV light on the pure copolymer without GO, as evidenced by the photothermal heating curve of pure copolymer aqueous solution (35 wt.%) under the present low-intensity UV (365 nm) irradiation conditions (Fig. S1 of Supplementary Information (SI)). We also found that it was easy to use both the thermal treatment around the body temperature and NIR light irradiation to make a sol-gel transition for both the pure copolymer and its nanocomposites with GO, attributed to the thermal-sensitivity of the amphiphilic copolymer (with sol-gel transition occurring at body temperature, *i.e.* around 37 °C^7^, as demonstrated in the phase diagram of the copolymer mPEG-PCL-mPEG; see Fig. S2 of SI) and to the appreciable thermal effect of the NIR light^28^, *e.g.*, even on pure water, as shown in Fig. S3 of SI. Although studies have demonstrated that rGO has a better photothermal effect as compared with GO, rGO can hardly be dispersed in an aqueous solution because of a lack of hydrophilic oxygen groups^15^,^29^,^30^. Without effective surface modification, rGO is ready to aggregate^31^,^32^, thus much lowing its performance for biomedical applications. Consequently, this study employs GO with fascinating solution properties to functionalize mPEG-PCL-mPEG, which can be easily dispersed in the polymer matrix. To rule out the possibility of the direct gelling of GO without involving the copolymer, an aqueous dispersion of GO at a concentration of 2.0 wt.% was also treated under the same UV conditions, and no gelling can be noted for the neat GO dispersion, as shown in Fig. S4 of SI. Nevertheless, even under such low-intensity UV irradiation, the GO structure is slightly changed in terms of restoring the π conjugation, as verified by a slight red shift of the typical UV/vis absorption band for GO (around 231 nm), as indexed to the π→π^*^ transition of aromatic carbon network (Fig. S5, SI)^33^. Actually, intense UV light irradiation (~1 W/cm^2^ that is around three magnitudes higher than that in our case) has been reported to convert GO to rGO, which is believed to be triggered by the photoinduced heating of the GO sheets, thus creating a high temperature and reactive environment localized at the sheets and their immediate aqueous medium^34^. To probe such a reactive environment that might also exist in our case despite the low-intensity UV, photodegradation of methylene (MB) dye with GO was carried out (Fig. S6, SI). We find that there are strong adsorption interactions between MB molecules and GO sheets, as demonstrated elsewhere^35^ and evidenced by our results that, only upon adsorption interactions for 23 h with UV off (*i.e.*, under dark conditions), the typical UV/vis absorption band of MB (around 660 nm) was largely decayed and shifts to about 670 nm. By contrast, in the absence of GO, MB shows a good stability against UV irradiation. Interestingly, it is noted that GO indeed has a photodegradation effect on MB, which can be confirmed by parallel tests (Fig. S6, SI); the one being subjected to the UV light irradiation corresponds to the resulting MB solution with a lower intensity of the characteristic UV/vis absorption band of MB. As a consequence, we believe that the present low-intensity UV illumination also enables the photoinduced heating of the GO sheets (as evidenced in Fig. S1 of SI) resulting from GO’s strong UV absorption, and exceptional thermal conductivity and specific heat capacity^15^, which is most likely to generate a thermal effect that facilitates the following UV light-induced sol-gel transition of the amphiphilic copolymer mPEG-PCL-mPEG.

**Figure 2:**
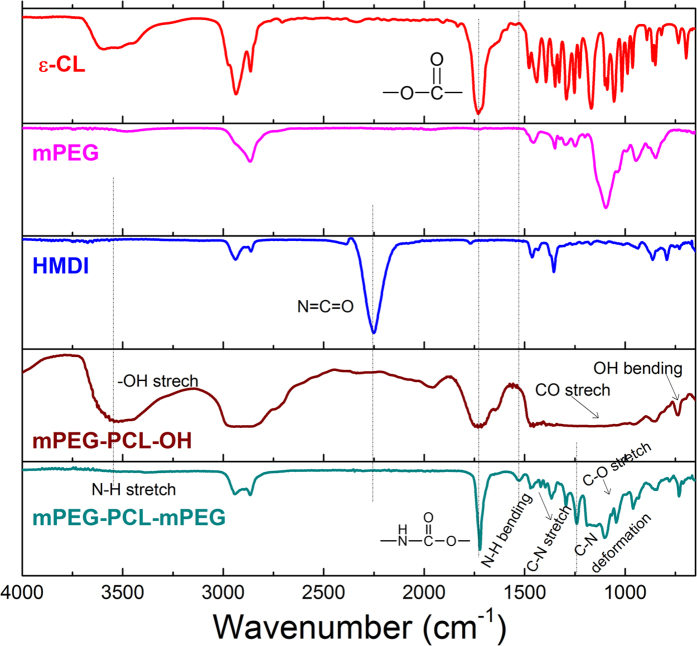
Structure detection of the triblock copolymer. ATR-FTIR spectra of the as-synthesized pure copolymer, intermediate product, and raw materials involved in the synthesis.

**Figure 3:**
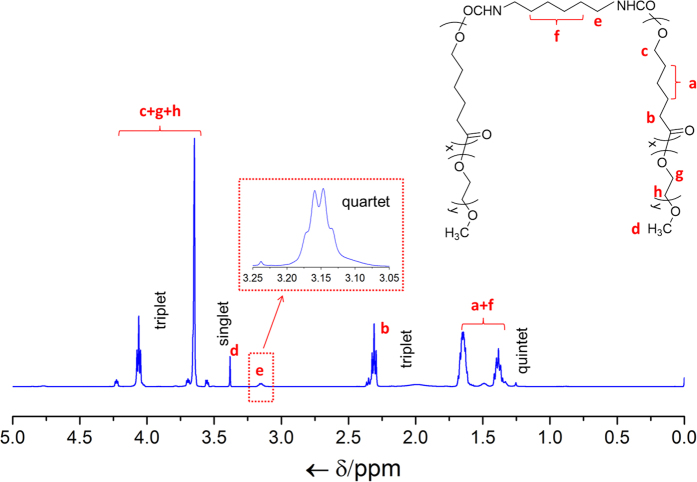
Structure confirmation of the triblock copolymer. ^1^H NMR spectra of the as-synthesized pure copolymer mPEG-PCL-mPEG.

The effective synthesis of the amphiphilic mPEG-PCL-mPEG is confirmed by ATR-FTIR and ^1^H NMR spectra, as shown in Figs 2 and 3 respectively. A ring opening polymerization of ε-CL was first conducted to synthesize an intermediate product mPEG-PCL-OH which was then coupled with HMDI, forming the final amphiphilic copolymer. The molecular structures used for showing the synthesis are presented in Fig. S7 (SI). As for ε-CL, a typical FTIR absorption at ~1729 cm^−1^ can be indexed to the lactone group. After polymerization with mPEG, the OH end group of mPEG-PCL-OH can be verified by the FTIR absorption around 3547, 1100 and 740 cm^−1^ assigned to the OH stretching, CO stretching and OH bending vibrations respectively^32^,^36^,^37^,^38^. After coupling mPEG-PCL-OH with HMDI, the NCO group (corresponding to the IR absorption at 2251 cm^−1^) of HMDI disappears, accompanied by the emergence of the amide group, as marked in Fig. 2, suggesting the effective coupling reaction and successful synthesis of the mPEG-PCL-mPEG^6^,^7^. To further ascertain the synthesis, the ^1^H NMR spectrum of mPEG-PCL-mPEG is provided in Fig. 3, together with the molecular structure displayed in the inset. The NMR absorption lines can be well indexed to the locations marked in the molecular structure.

**Figure 4:**
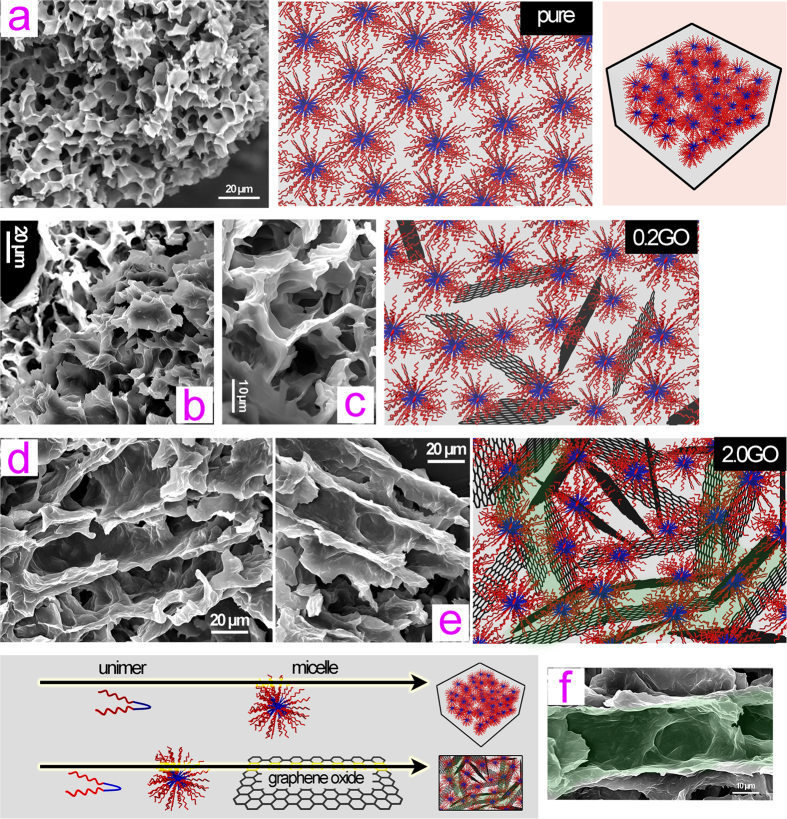
Microstructure observation. SEM observation of the microstructures of lyophilized gel samples including pure copolymer-based thermogel (**a**) and UV-gels 0.2GO (**b,c**) and 2.0GO (**d**–**f**). The corresponding schematic illustration of the microstructures of the lyophilized gel samples is also presented on the right side, with light green shadings to highlight the microchannels within the nanocomposite 2.0GO.

After confirming the effective synthesis of the copolymer mPEG-PCL-mPEG, we then performed a SEM study of the microstructure of the lyophilized UV-gel (Fig. 4). For comparison, the pure copolymer gel was also prepared by a body temperature-induced so-gel transition. All the hydrogel samples exhibit microporous structures. The micropores of the pure copolymer gel are shown to be more uniform, with a smaller size, as compared to those of the composite gels 0.2GO and 2.0GO. This is because the prepared GO with a large lateral size (at microscale) heavily disturbs the aggregation of the polymer micelles in an ordered fashion. The structural details of the GO are presented in SI by means of various characterizations including SEM (Fig. S8), AFM (Fig. S9), XRD (Fig. S10) and FTIR (Fig. S11), which is good line with the GO structure as reported elsewhere^13^,^39^,^40^. The oxygen groups of the GO sheets (Figs S10 and S11, SI) are also likely to have an interaction with the hydrophilic blocks of mPEG-PCL-mPEG, which may exert an disruption impact on the hydrophilic and hydrophobic balance between mPEG and PCL blocks, despite the fact that the FTIR spectra of all these samples (*i.e.* pure polymer, 0.2GO and 2.0GO) are close to each other, with only a slight difference (*e.g.* the IR absorption stemming from the NH stretches), as shown in Fig. S12 (SI). It is particularly interesting to observe the microchannels within the composite gel with a higher GO concentration, in this case 2.0GO (Fig. 4d,f). The large size of GO sheets, as well as the existence of both hydrophilic and hydrophobic domains on their surface, most likely makes it ready to form these microchananels, as also depicted by a structural model in Fig. 4 (light green shadings are used to highlight some microchannels). Such microchannels are believed to be useful for controlled drug release applications, since their presence can accelerate the sustained drug release through reducing the drug diffusion barrier as a result of a microchannel conducting effect^22^,^23^,^24^,^41^.

**Figure 5:**
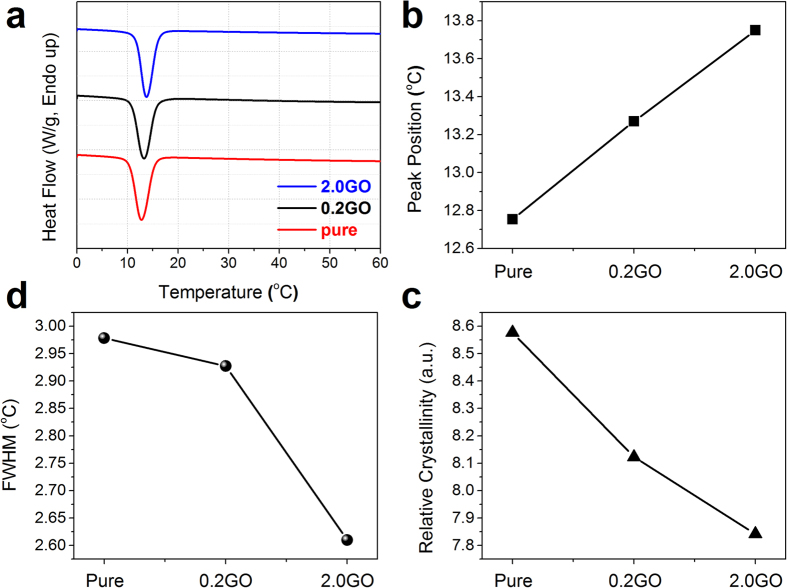
Thermal analysis. (**a**) The 2^nd^-run cooling thermogram for different samples. (**b,c,d**) Comparison of the crystallization peak position (**b**) relative crystallization (**c**) and FWHM (**d**) among different samples. Note that the relative crystallization was calculated as the crystallization peak area (unit: J/g).

DSC is also employed to further demonstrate the molecular structure of the copolymer-based nanocomposites (Fig. 5 and Figs S13–S15, SI). All the samples exhibit two melting peaks that can be found on the 2^nd^–run DSC heating curves (Figs S13–S15, SI), while only one crystallization peak can be seen on the 2^nd^–run DSC cooling thermograms (Fig. 5). The appearance of the double melting peaks is related to the recrystallization of PCL blocks during the heating process^42^,^43^. The crystallization peak temperature is stepwise increased from pure copolymer, 0.2GO, to 2.0GO (Fig. 5(b)), whereas FWHM (Fig. 5(c)) and relative crystallization (Fig. 5(d)) show the opposite trend. The incorporation of GO may have a nucleation effect on the crystallization of part of polymer chains around GO sheets, which thus leads to the quicker crystallization during the DSC cooling process. However, the presence of GO also has a disturbing impact on the overall regular packing of copolymer chains, thereby decreasing the relative crystallization and FWHM of the resulting composites. It is reasonable to observe that the higher content of GO can make the nucleation and disturbing effects more striking (Fig. 5).

**Figure 6:**
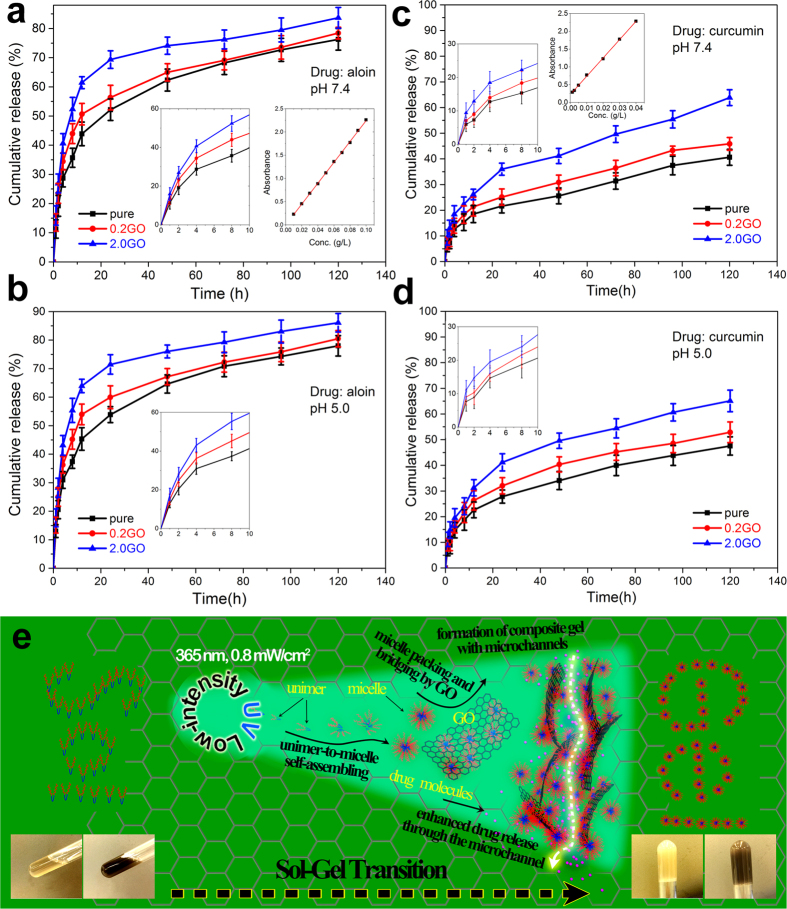
Controlled drug release performance evaluation. The controlled drug release test results for aloin at pH 7.4 (**a**) and pH 5.0 (**b**) and for curcumin at pH 7.4 (**c**) and pH 5.0 (**d**). (**e**) Schematic illustration of the present UV-triggered sol-gel transition and microchannel formation within the amphiphilic copolymer-based UV-gel filled with GO sheets, as well as of the microchannel conduction effect on the accelerated drug release. The inset on the left side of each panel is a selectively magnified to show the initial 10 h drug release behavior; the right side one is the calibration curve of the corresponding drug.

The sustained drug release performance of the hydrogel samples was evaluated by an *in vitro* study. We employed aloin and curcumin as the hydrophilic and hydrophobic model drugs respectively, with the results presented in Fig. 6(a–d). To mimic the physiological pH of blood and the acidic intracellular environment in tumor cells (attributed to the metabolic end products accumulate in the tumor microenvironment^44^), pH 7.4 and pH 5 buffer solutions were employed respectively^45^,^46^. Both aloin and curcumin loaded in the three hydrogel samples display typical drug release profiles, *i.e.* initial rapid release and then sustained release (like a parabola trace over the entire releasing period). Under pH 7.4 conditions, the final releasing contents of aloin and curcumin are calculated to be 70~85% and 40~65% respectively after 120 h, as shown in Fig. 6(a,c) respectively. In comparison with the pH 7.4 conditions, it can be noted that the drug release rate of the same drug carrier is higher in the pH 5 buffer for both the drugs, in this case aloin (Fig. 6(b)) and curcumin (Fig. 6(d)). Bar plots of comparing the cumulative release contents after the 24 h of release are also depicted in Fig. S16 of SI. We speculate that the higher release rate in the acidic buffer solution can be ascribed to the following points according to the literature^45^,^47^,^48^,^49^: (i) the amide and ester groups of the copolymer mPEG-PCL-mPEG become more hydrolysable under acidic conditions, thus indicating that the copolymer-based micelles are likely to be somewhat disrupted^47^; (ii) the higher solubility of the drugs in the acidic solution^49^; (iii) protonation of drug molecules in the acidic solution could weaken their interactions with the drug vehicle to some extent^45^. Consequently, such a release characteristic of our drug carrier, namely the increased release rate under acid conditions of tumor microenvironment, will hold great promise in the enhanced treatment of patients with malignant tumor, cancer and other diseases.

As expected, the GO inclusion drives the release of both the drugs. The higher the GO concentration, the faster the drug release rate. This can be well explained by the GO-induced microchannel formation (as confirmed by SEM)^41^. More GO incorporated results in the generation of more microchannels that could play a conducting role for the drug release out of the confining network of the hydrogel and hence reduce the diffusion barrier against the drug molecules. Such a microchannel conduction effect on the sustained drug release is also schematically illustrated in Fig. 6(e), along with the depiction of the sensitive sol-gel transition under the present low-intensity UV conditions. We also note that the hydrophobic drug (curcumin) is most likely to possess a higher stability within the network structure of the hydrogel, thereby leading to a lower release concentration, relative to the case of the hydrophilic drug (aloin) at the same time interval. To further explain the difference, we turn to discussing the copolymer-based micelle structure. The core-shell structure of the micelle formed in the aqueous solution consists of hydrophilic mPEG blocks as the shell and hydrophobic PCL blocks as the core. As a result, the hydrophilic drug should locate around the shell, while the hydrophobic drug are readily driven into the core^50^. Therefore, the hydrophobic drug can be better stabilized under the aqueous buffer conditions.

Cytotoxicity is one of the most important properties of biomaterials, we thus further investigate the cytotoxicity of our prepared copolymer-based biomaterials incorporating with GO, despite the fact that our previous investigation has shown that the pure copolymer mPEG-PCL-mPEG, basically, shows non-cytotoxicity^7^. It is also satisfying to find that the incorporation of GO into the copolymer can only negligibly change the cytotoxicity of the copolymer on the basis of tetrazolium-based colorimetric (MTT) and lactate dehydrogenase (LDH) assays. Specifically, as revealed in Fig. S17(a) of SI, the positive control exhibits the lowest percentage of cell viability, while the negative control shows the highest. Also note that the copolymer-based samples have a very low toxicity to cells, rather close to the negative control, although more GO incorporated can increase the cytotoxicity to a limited extent as compared to the pure copolymer. Regarding the LDH assay (Fig. S17(b) of SI), a high LDH release is an implication of the cell membrane damage. As expected, the positive control shows the highest LDH release, in great agreement with the MTT assay results. The other samples including negative control, pure copolymer, 0.2GO, and 2.0GO show a very limited effect on the LDH release despite the fact that the GO incorporation can afford little increase of cytotoxicity to the copolymer. Consequently, the results of the combined MTT and LDH assays demonstrate that our prepared copolymer and its composites with GO are basically non-cytotoxic.

## Discussion

The high sensitivity of the copolymer-based physical gel filled with GO, which is responsive to the low-intensity UV at 365 nm, can be conjectured as the following aspects: (1) exceptional photothermal properties of GO, such as strong 365 nm UV absorption properties, bring UV responsiveness to the copolymer; (2) strong interactions between the copolymer-based micelles and GO sheets enable the photothermal effect generated by GO to be efficiently transferred onto the thermosensitive copolymer; (3) GO sheets are most likely to have a bridging effect on the micelles, expediting the aggregation of the micelles and hence the consequent gelation, and (4) introduction of a new network structure made of GO sheets into the network structure constructed by the copolymer-based micelles highly sensitizes the gelation based on aggregation of the micelles.

The mechanism for the GO-induced microchannel formation is discussed as the following considerations: (i) the large surface area of GO lays the foundation of the microchannel; (ii) the abundant functional groups on GO surfaces make GO sheets have strong interactions with the mPEG-PCL-mPEG copolymer-based micelles, which disturbs the regular packing of the micelle (regular micelle packing gives uniform micropores of the thus-obtained pure copolymer-based gel sample; see Fig. 4); (iii) the copolymer-based micelles pack along the GO sheets, leading to the formation of microchannels. Considering the GO-enabled acceleration of drug release, the conducting effect of microchannels can be considered as the main reason, as well presented in the literature^22^,^23^,^24^,^41^.

The unique characteristics and advantages of the present amphiphilic copolymer-based nanocomposites incorporating with GO are discussed as follows: (i) the preparation of the nanocomposites is easy to realize through a facile aqueous solution method by virtue of the fascinating solution properties of GO; (ii) a low extent of GO incorporation (in this case 0.2 wt.%) can highly sensitize the amphiphilic copolymer with respect to achieving a low-intensity UV light-driven sol-gel transition which cannot be realized if GO is absent; (iii) the inclusion of the GO, even at a high content (in this case 2.0 wt.%), has a negligible effect on the non-cytotoxicity of the copolymer (the prepared pure copolymer and its nanocomposites with GO at both higher and lower contents, basically, exhibit non-cytotoxicity); (iv) the GO can induce the formation of significant microchannels within the amphiphilic copolymer-based composite gels, which reduces the diffusion barrier of the drug molecules and hence accelerates the sustained drug release, as a result of the microchannel conducting effect, and (v) the GO-incorporated amphiphilic copolymer system shows high potential for achieving enhanced photothermal therapy and chemotherapy for the treatment of patients with cancer and other diseases, by virtue of the graphene chemistry (such as remarkable photothermal properties) and amphiphilic copolymer-based sol-gel transition with an enhanced sensitivity.

In summary, highly sensitive, non-cytotoxic mPEG-PCL-mPEG-based nanocomposites with GO have been prepared, which show a high sol-gel transition sensitivity to a low-intensity UV light. Significant microchannels are formed within the UV-gel, which enable an increase of the sustained drug release rate through reducing the diffusion barrier for drug molecules. The present study paves the way for the fabrication of various amphiphilic copolymer-based composites/nanocomposites incorporating diverse light-sensitive materials, which shows potentials for enhanced photothermal therapy and chemotherapy in biomedical fields.

## Methods

### Materials

Stannous octoate (Sn(Oct)_2_), mPEG (Number-average molar mass (Mn) 550), ε-caprolactone (ε-CL), hexamethylene diisocyanate (HMDI), curcumin, and aloin were purchased from Sigma-Aldrich and used without further purification. All other reagents were used as obtained until otherwise stated.

### Synthesis of the pure copolymer mPEG-PCL-mPEG

Typically, 0.1 mol of ε-caprolactone (ε-CL), 0.01 mol of mPEG, and 0.3 wt.% of stannous octoate (Sn(Oct)_2_) were added into a reaction vessel under dry nitrogen atmosphere, which was kept at 130 °C for 12 h and then cooled to 80 °C. Afterwards, 0.01 mol of hexamethylene diisocyanate (HMDI) was added, and the resulting mixture was stirred at 80 °C for 3 h, followed by degassing under vacuum for 1 h. Finally, the resultant copolymer was obtained after cooled to room temperature (25 °C). The calculated average molecular weight of the synthesized copolymer mPEG-PCL-mPEG is presented in Table S1 of SI.

### Preparation of pure copolymer-based hydrosol and thermogel

The as-synthesized copolymer was added into deionized (D.I.) water, forming a copolymer solution at a 20 wt.% concentration. The mixture was then vigorously agitated mechanically at 60–70 °C, which resulted in the formation of a white milky viscous liquid. Next, the reaction vessel was placed into an ice bath for cooling the polymer solution, producing the final transparent polymer sol. To fabricate a pure copolymer-based thermogel, the prepared sol was thermally treated at body temperature to induce a sol-gel transition.

### Preparation of the GO-copolymer composite sols

GO was prepared by a modified version of Hummers’ method as reported elsewhere^32^,^39^. Two aqueous dispersions with GO concentrations of 0.2 and 2.0 wt.% were firstly prepared by 1 h sonication treatment (150 W). After that, the as-synthesized copolymer was added to the GO dispersions (the weight ratios of GO and copolymer were kept at 0.2/20 and 2.0/20, with the copolymer concentration fixed at 20 wt.%). The mixture was then vigorously agitated at 60–70 °C. Afterwards, the reaction vessel was placed into an ice bath to cool the dispersion, and finally the sols with different weight loadings of GO were obtained.

### UV light-driven sol-gel transition of the GO-copolymer composite sols

The as-prepared composite sol at a given amount were placed into a glass tube which was then placed inside a UV lamp box (the lamp used has an irradiation wavelength of 365 nm). The UV light intensity reaching the surface of the sol in the glass tube was detected to be 0.8 mW/cm^2^ by a UVA light intensity detector. After 10 min irradiation, the light was turned off, and the formation of a gel was recorded by a test tube inverting method. The prepared composite hydrogels were labeled as 0.2GO (GO/copolymer: 0.2/20) and 2.0GO (GO/copolymer: 2.0/20). The corresponding dried gel samples were prepared by lyophilization for 24 h, which were used for the following various characterization measurements. For comparison, the pure copolymer sol was also irradiated under UV light with the same above-described conditions.

### Characterization

The FT-IR spectra were recorded on a Perkin Elmer paragon 1000 infrared spectroscope using an attenuated total reflection (ATR) mode with a scanning range of 4000–650 cm^−1^. ^1^H NMR spectrum was captured on a Varian Unity Inova 500 NB NMR Spectrometer using CDCl_3_ as the solvent. DSC was carried out with a PerkinElmer DSC 8000 differential scanning calorimeter. Scanning electron microscopy (SEM, a TM3000 Tabletop Microscope, Hitachi) was used to observe the microscopic morphologies of the pure block copolymer hydrogel, and the composite hydrogels 0.2GO and 2.0GO. The specimens employed for the SEM observation were prepared by pre-freezing the wet hydrogels in liquid nitrogen and then lyophilization for 24 h. Prior to the SEM observation, a thin gold film was sputtered onto the lyophilized specimen surface. UV/vis spectra were recorded on a Lambda 18 UV/vis spectrometer. Waters 1515 gel-permeation chromatography (GPC, Waters Co., Milford, USA) was employed to determine the macromolecular weight and macro-molecular weight distribution of the prepared copolymer mPEG-PCL-mPEG.

### The *in vitro* skin toxicity test

The toxic effects of the hydrogel sample on skin were determined by MTT and LDH assays, with 5% (w/v) sodium dodecyl sulfonate as the positive control and the non-treated skin insert as the negative control.

#### Epiderm culture

A commercially available human epidermal equivalent, that is the epiderm (EPI-200, MatTek Corporation, Ashland, MA, USA), was employed to mimic normal human epidermis. These epiderm cultures were comprised of human-derived epidermal keratinocytes, which were cultured on standing cell culture inserts (Millipore, Billerica, MA, USA) at the air–liquid interface to form a multilayered and differentiated model of the human epidermis. The epiderm cultures were placed in 6 wells plates and then pre-conditioned overnight at 37 °C under 5% CO_2_. Test samples were added to the skin inserts the next day and allowed to incubate for 1 h. Afterwards, all skin inserts were transferred to a fresh medium for the release of cytokines to the medium for 24 h. The medium was recovered and stored at −80 °C for the LDH assay. All skin inserts were then transferred to fresh plates for the MTT assay.

#### The LDH assay–cell membrane integrity test

The integrity of cell membrane (or cell damage) of the epiderm cultures was measured by a colorimetric LDH assay (LDH Cytotoxicity Detection Kit, Takara Bio Inc., Otsu, Shiga, Japan). This assay measures the membrane integrity as a function of the amount of cytoplasmic LDH being released into the medium. Briefly, an assay mixture was prepared by mixing one portion of the LDH assay catalyst with 45 portions of dye solutions. For all cultures, the assay mixture was added to the medium in equal proportions. After incubation for 30 min at room temperature under dark conditions, the coloration reaction was stopped with HCl (1.0 M). A plain medium was used as a control in this assay. Absorbance and background correction were performed at 490 nm. The cell membrane integrity was expressed as the ratio of the amount of LDH released (per treatment) to the maximum amount of LDH released from the positive control.

#### The MTT assay–cell viability test

Skin inserts were transferred to fresh plates with a pre-filled MTT solution (MTT was dissolved in phosphate buffered saline (PBS) at 5 mg/mL and filtered to sterilize and remove a small amount of insoluble residue being present in some batches of MTT) and were allowed to incubate at 37 °C under 5% CO_2_ for 3 h. Upon completion of incubation, all MTT solutions were removed. Skin inserts were transferred to fresh plates, and isopropanol was added to each insert for 2 h for extraction of formazan, before transferring to 96 wells plates for the spectrophotometric analysis at 550 nm. Cytotoxicity was expressed as the ratio of cell viability (per treatment) to the maximum cell viability, as calculated from the negative control.

### *In vitro* drug release behavior study

Aloin and curcumin were selected as hydrophilic and hydrophobic model drugs respectively for the controlled drug release study. In the case of hydrophilic drug (aloin), the hydrogels, loaded with aloin (0.1% w/w%), were immersed in a 20 mL PBS (pH 7.4 or pH 5) solution and incubated at 35 ± 2 °C in a shaking water bath at 50 rpm for specific time intervals (1, 2, 4, 8, 12, 24, 48, 72, 96, 120 h). The drug release buffer solution was carefully withdrawn and filtered through a filter paper, and the filtrate was analyzed with a UV–vis spectrophotometer. The characteristic UV/vis absorption at *λ*_max_ = 291 nm was used to monitor the release behavior of the aloin. The accumulated drug release was then calculated. The experiment was performed in triplicate, with the standard deviation calculated as the error bar in the drug release profile. Following a test at a time interval, the obtained filtrate and residue were put back into the sustained drug release solution, so as to keep the drug release system at a steady state. The calculation of the accumulated release is given as the following equation.


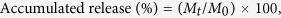


where *M*_*t*_ is the amount of the drug being released from the hydrogel at the time *t,* and *M*_*0*_ is the initial amount of the drug being loaded in the hydrogel.

As for the hydrophobic model drug, namely curcumin, the procedures of the sustained drug release test was described as follows: a given amount of curcumin (0.1% w/w%)-loaded hydrogel was coated onto a glass sheet after the sol-gel transition. Subsequently, the coated glass sheet was immersed into a 20 mL PBS (pH 7.4) solution which was then mechanically stirred at 50 rpm and 35 ± 2 °C. At specific time intervals, namely 1, 2, 4, 8, 12, 24, 48, 72, 96 and 120 h, aliquots were withdrawn with a pipette, followed by filtering through a filter paper. The obtained filtrate was then analyzed by the UV–vis spectrophotometer, and the characteristic UV/vis absorption at *λ*_max_ = 426 nm was used to monitor the curcumin release behavior. The accumulated drug release was then calculated. The results were presented as the cumulative release as a function of release time, and the experiment was performed in triplicate.

## Additional Information

**How to cite this article**: Hu, H. *et al*. Graphene oxide-enhanced sol-gel transition sensitivity and drug release performance of an amphiphilic copolymer-based nanocomposite. *Sci. Rep.* **6**, 31815; doi: 10.1038/srep31815 (2016).

## Supplementary Material

Supplementary Information
