# Supplementary material for: Graphene oxide-enhanced sol-gel transition sensitivity and drug release performance of an amphiphilic copolymer-based nanocomposite
Source: Sci Rep. 2016 Aug 19;6:31815. doi: 10.1038/srep31815 (PMC4990926; doi:10.1038/srep31815)
Supplement: Supplementary Information [file srep31815-s1.pdf]

Supplementary Information

Graphene oxide-enhanced sol-gel transition sensitivity and drug release performance of an amphiphilic copolymer-based nanocomposite

Huawen Hu[1], Xiaowen Wang[2], Ka I Lee[2], Kaikai Ma[2], Hong Hu[2] & John H. Xin[2]

[1]Foshan University, Guangdong, 528000, China. [2]The Hong Kong Polytechnic University, Hong Kong SAR, 999077, China. Correspondence and requests for materials should be addressed to H.W.H. (email: huhuawen2664@sina.com), J.H.X. (email: tcxinjh@polyu.edu.hk) or H.H (email: tchuhong@polyu.edu.hk)

This PDF file includes:

Figures S1 to S17

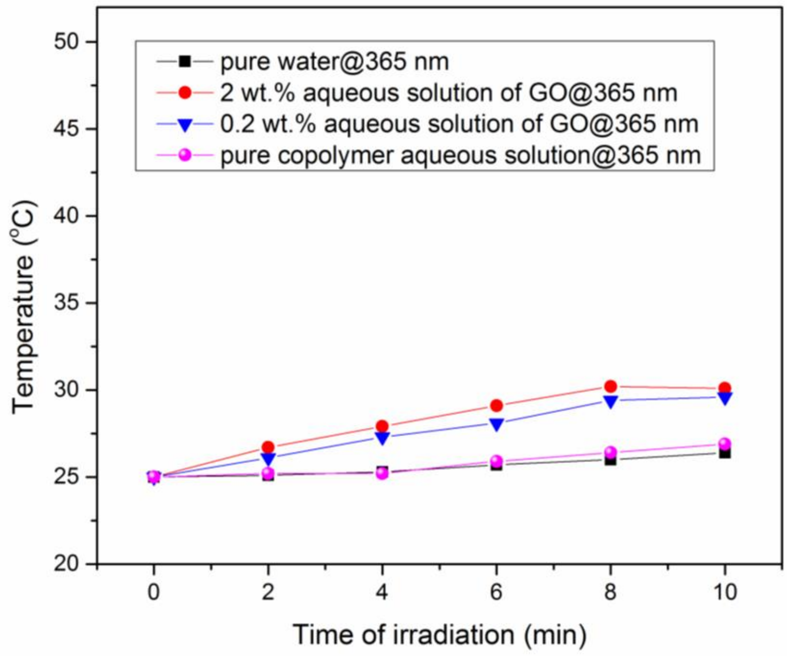

Figure S1. Low-intensity UV-based photothermal heating curves of pure water, 0.2 wt.% aqueous solution of GO, 2.0 wt.% aqueous solution of GO, and pure copolymer aqueous solution (35 wt.%).

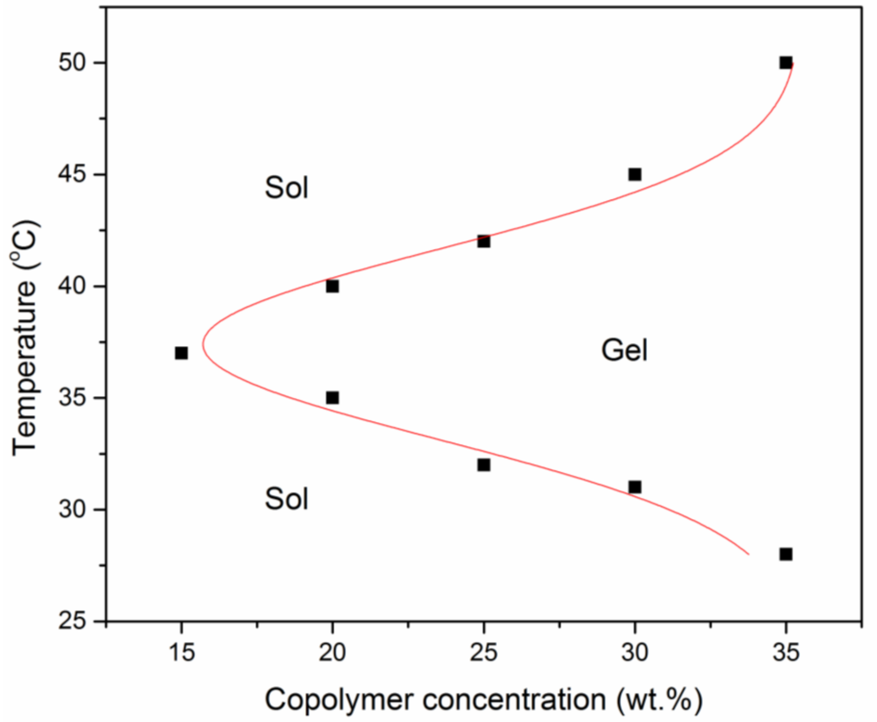

Figure S2. The phase diagram of the copolymer mPEG-PCL-mPEG.

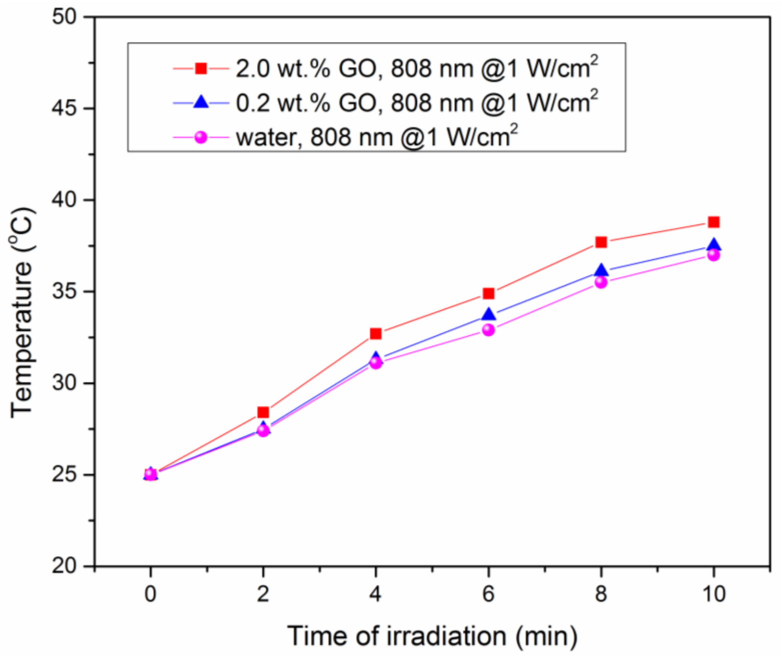

Figure S3. NIR light-based photothermal heating curves of pure water, 0.2 wt.% aqueous solution of GO, and 2.0 wt.% aqueous solution of GO.

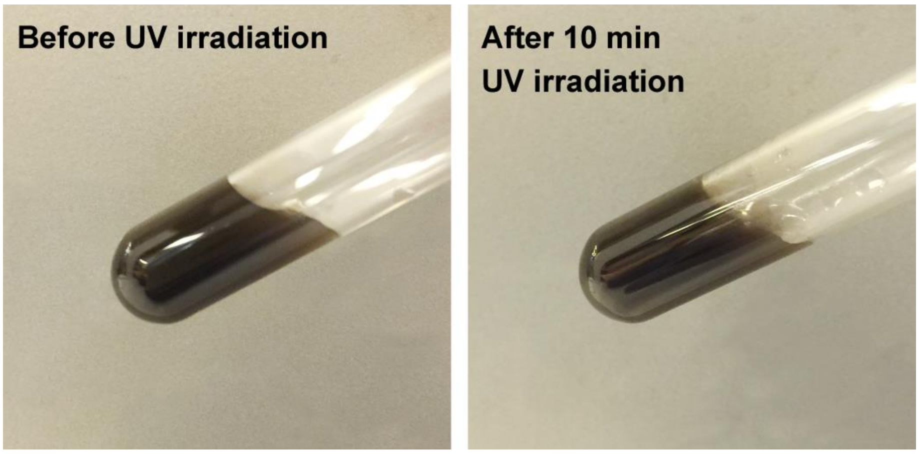

Figure S4. Photoimages showing 2.0 mg/mL aqueous GO dispersions before and after 10 min UV irradiation (λ= 365 nm, UV light intensity: 0.8 mW cm^{-2}).

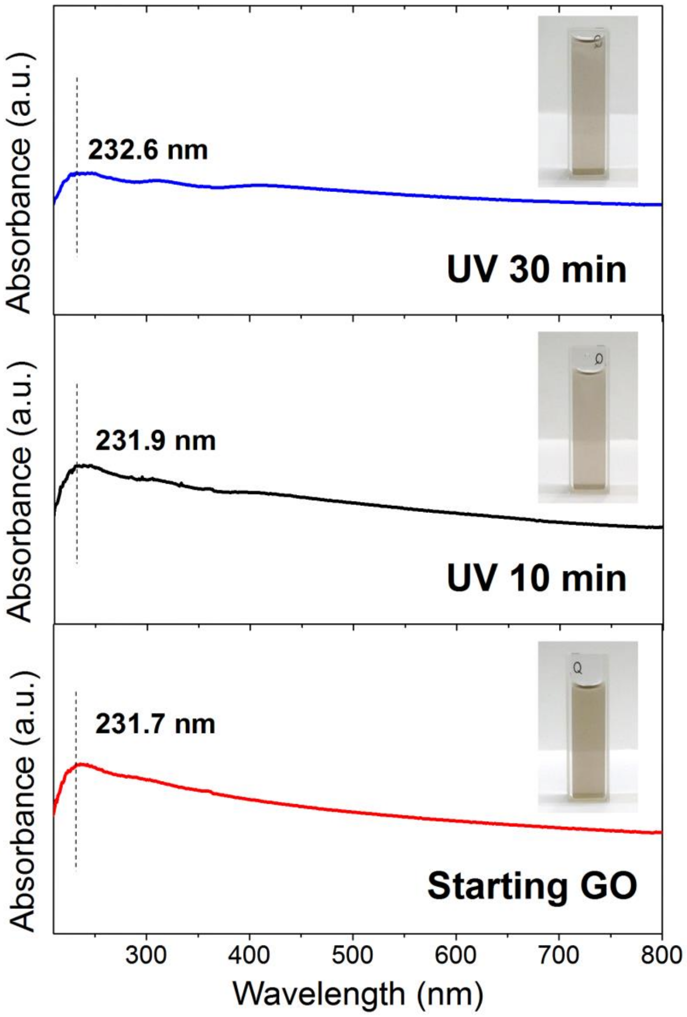

Figure S5. The UV/vis spectra of diluted water dispersions of the pristine GO, and the GO treated by UV for 10 and 30 min. The insets show the corresponding digital images of the diluted water dispersions.

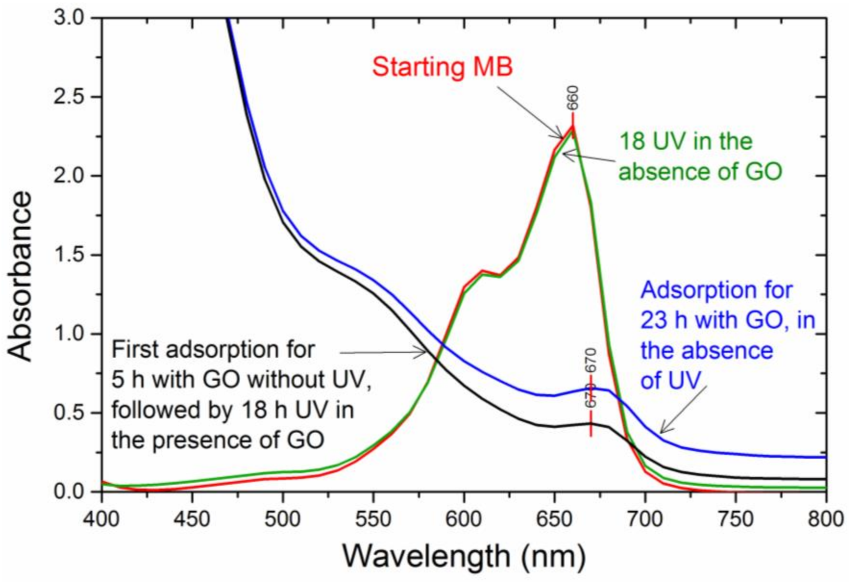

Figure S6. Investigation of photodegradation of MB dye with GO under UV light irradiation (Conc. of GO: 2.0 wt.%, Conc. of MB: 10 mg/L, UV@365 nm: 0.8 mW/cm^2). Only under UV (in the absence of GO), there is a negligible influence on the MB dye (see green line *vs.* red line that corresponds to the starting MB solution). On the other hand, only with GO (in the absence of UV), obvious adsorption interactions between the MB dye and GO can be noticed, leading to a distinct decrease of the characteristic UV/Vis adsorption band of MB after 23 h adsorption interaction under dark conditions (see blue line *vs.* the starting red line). Most importantly, we find that GO has a photocatalytic degradation effect on the MB dye in view of the fact that, under both UV and GO conditions (5 h adsorption interactions with GO under dark, combined with 18 h interactions with GO under UV), the intensity of the characteristic UV/Vis adsorption band of MB is the lowest among all the cases considered (see black line vs. all other lines).

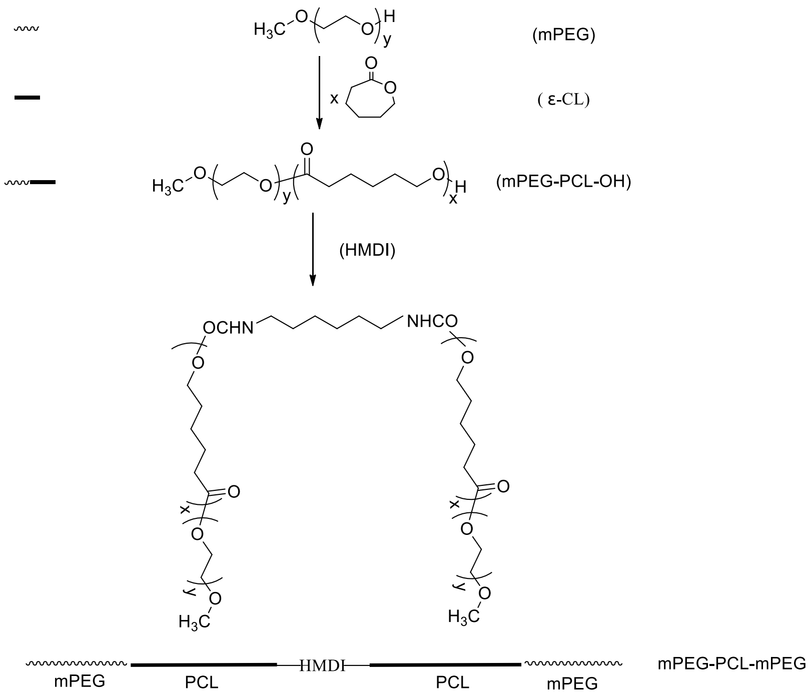

Figure S7. Schematic diagram, with molecular structures, showing the synthesis of the block copolymer mPEG-PCL-mPEG.

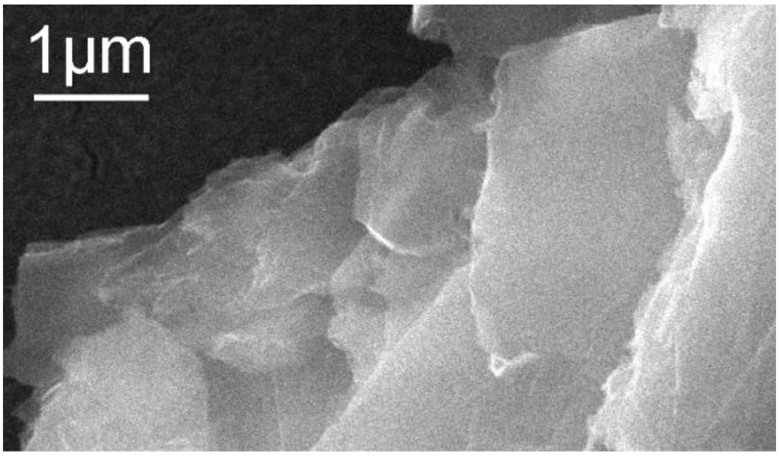

Figure S8. SEM image of GO.

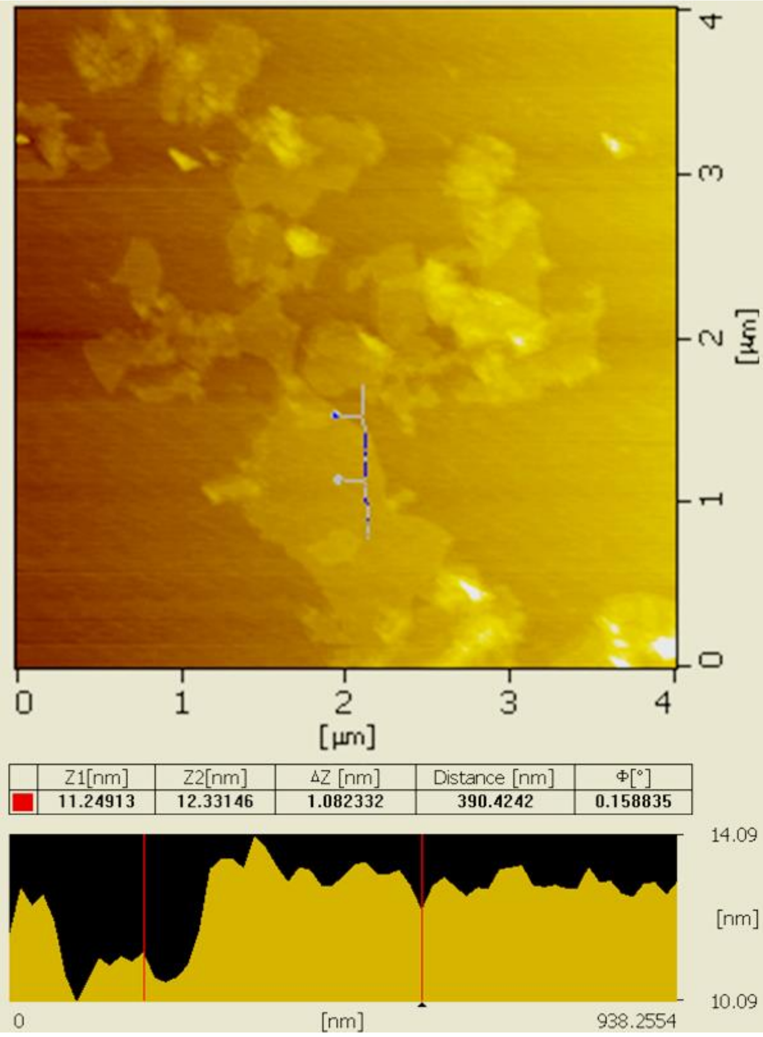

Figure S9. AFM image of GO.

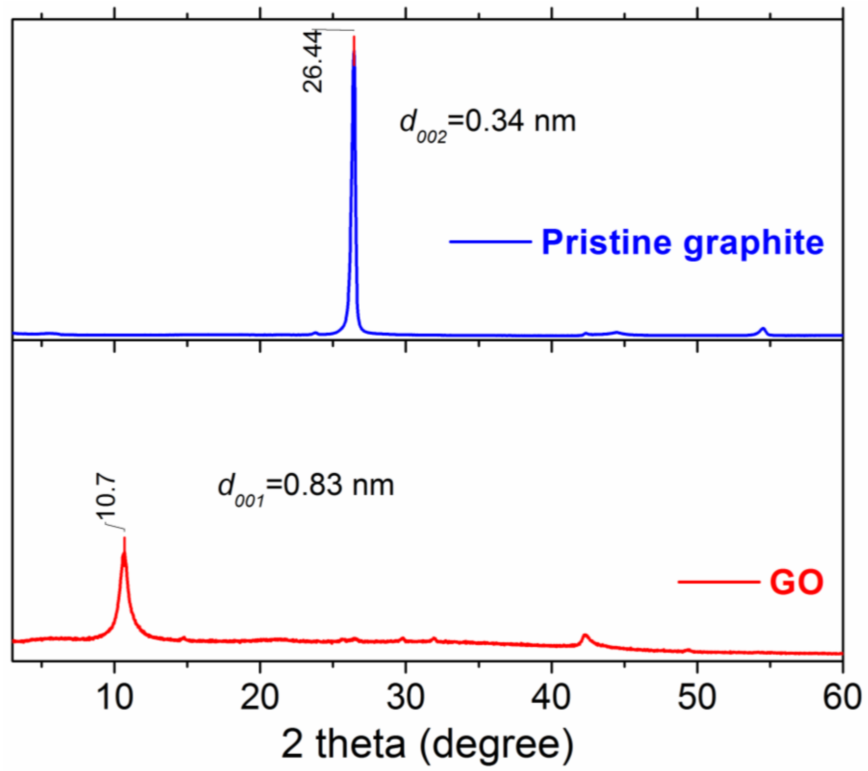

Figure S10. XRD patterns of the pristine graphite and GO. GO shows a much larger interplanar spacing (0.83 nm) as compared to the pristine graphite (0.34 nm), on the basis of Bragg's Law. This is attributed to the incorporation of abundant oxygen groups on the GO planes, which make GO hydrophilic and highly dispersible in water media.

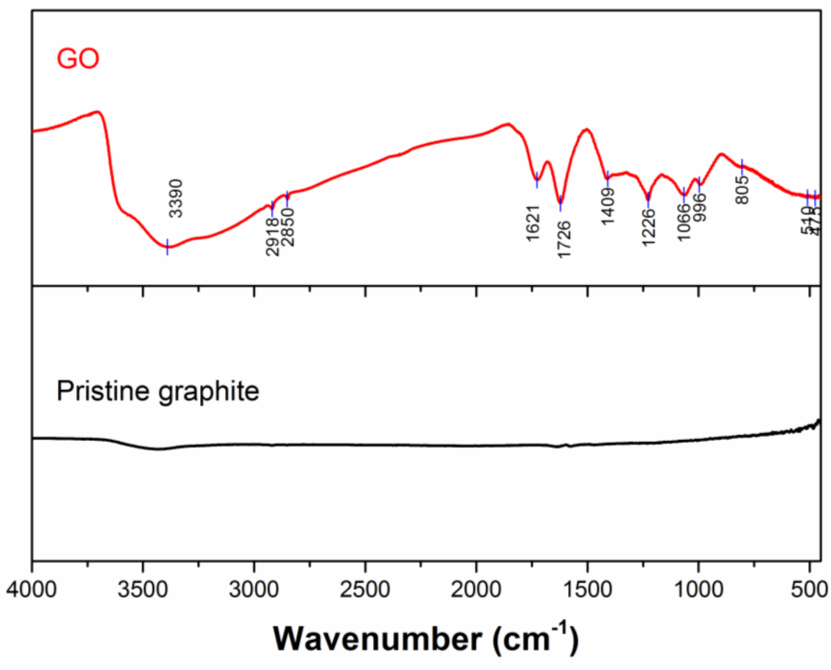

Figure S11. FTIR spectra of the pristine graphite and GO. A plenty of oxygen groups have been incorporated onto GO planes after the effective oxidation of graphite, as confirmed by the significantly stronger FTIR absorption bands indexed to different types of oxygen groups, along with the higher extent of sp^3 hybridization in the GO planes.

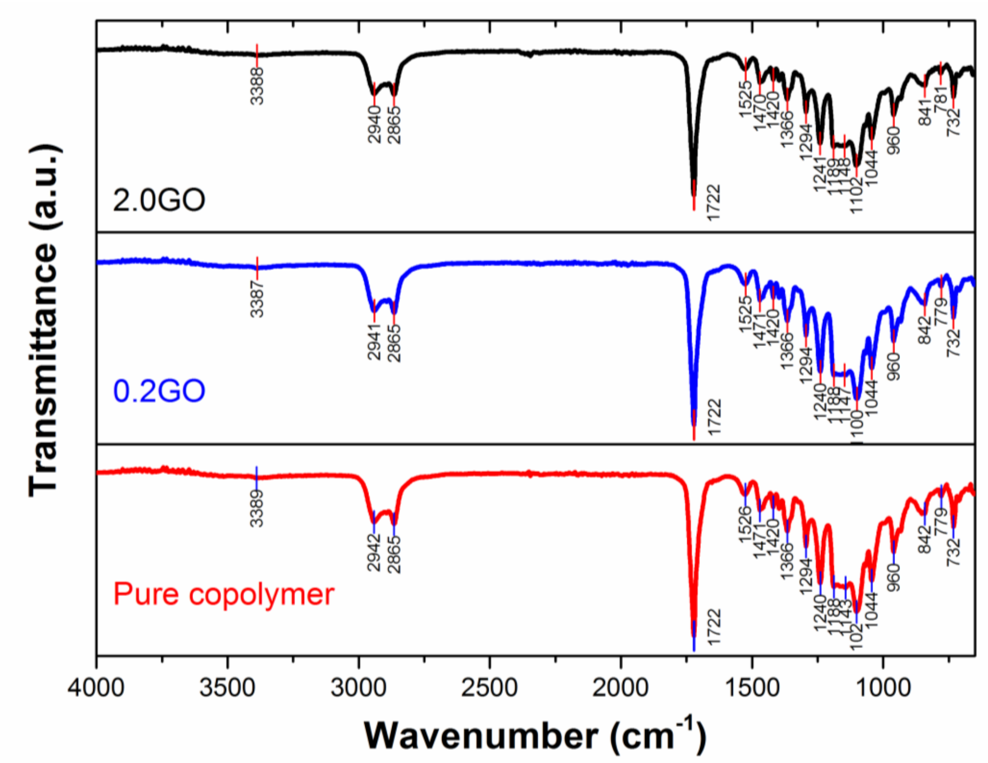

Figure S12. ATR-FTIR spectra of the pure copolymer mPEG-PCL-mPEG, and copolymer-based composites 0.2GO and 2.0GO.

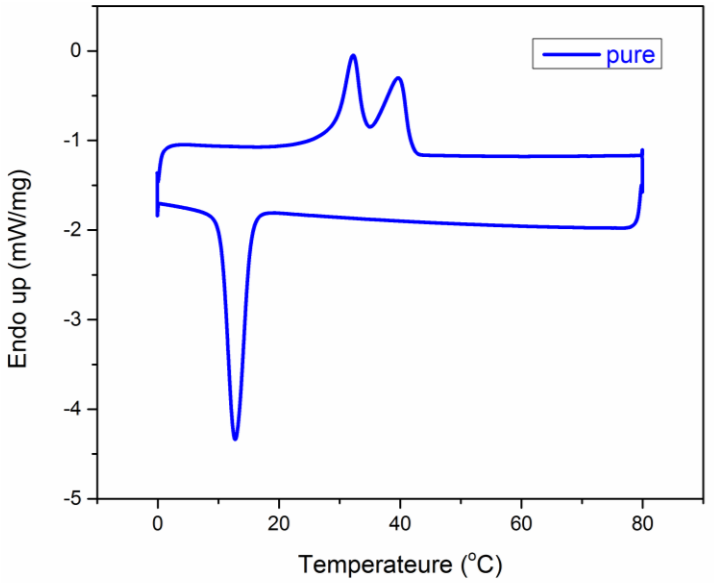

Figure S13. DSC thermograms of the pure copolymer, including the 2nd-run heating and 2nd-run cooling thermograms after the removal of the thermal history via the 1st-run heating and cooling processes.

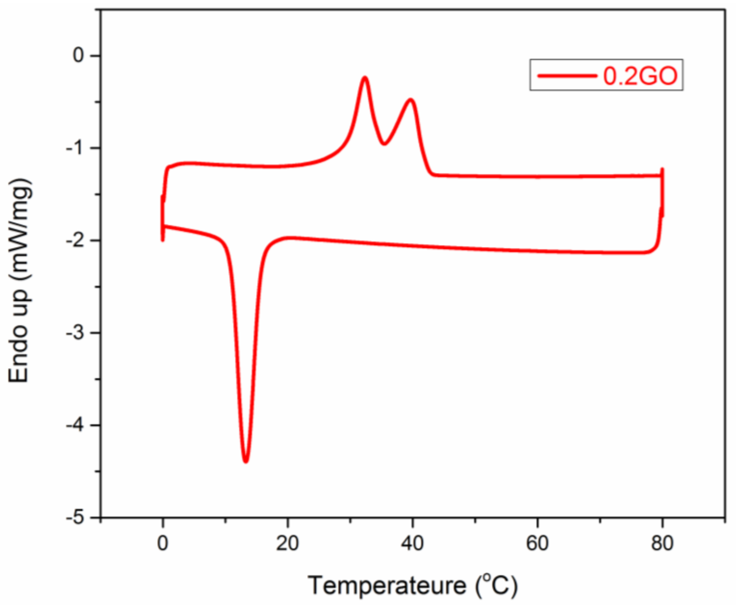

Figure S14. DSC thermograms of 0.2GO, including the 2nd-run heating and 2nd-run cooling thermograms after the removal of the thermal history via the 1st-run heating and cooling processes.

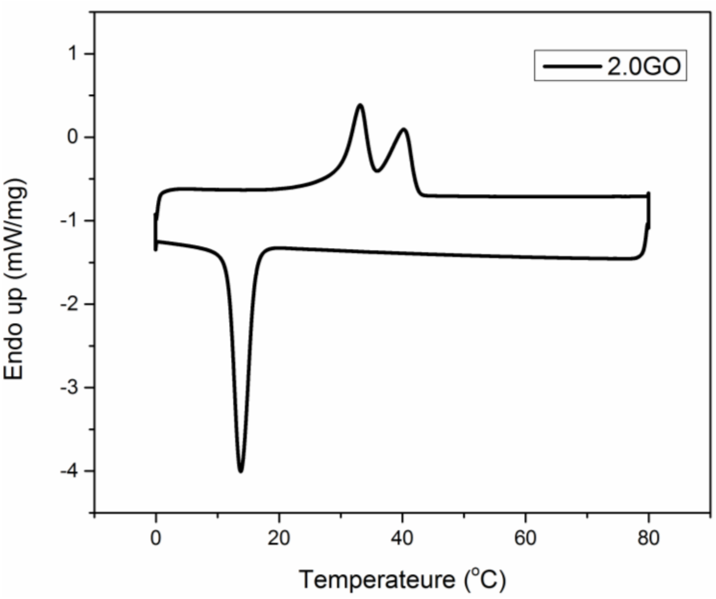

Figure S15. DSC thermograms of 2.0GO, including the 2nd-run heating and 2nd-run cooling thermograms after the removal of the thermal history via the 1st-run heating and cooling processes.

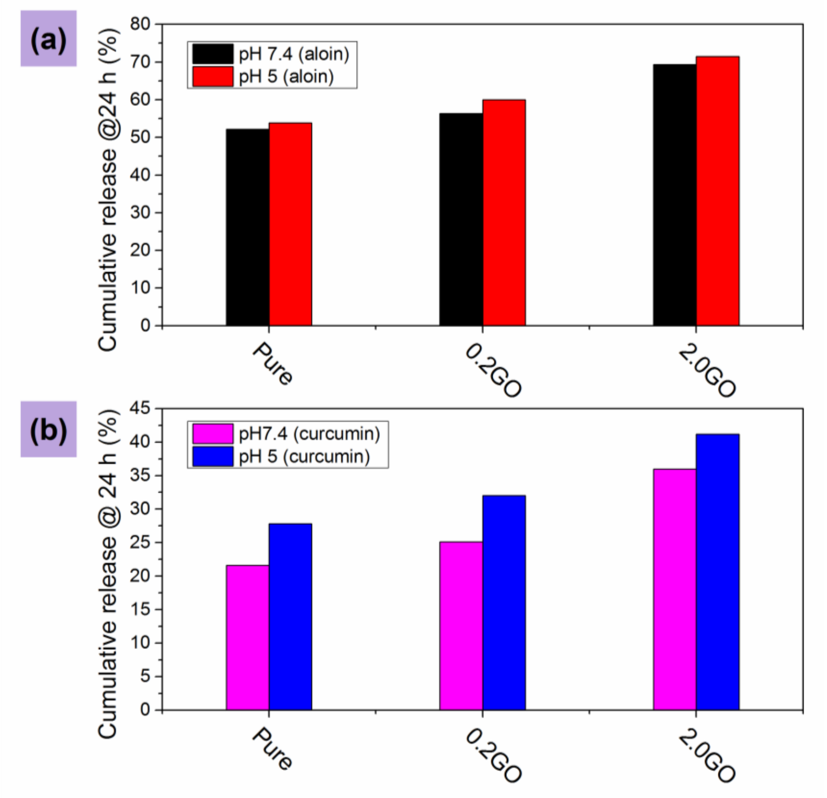

Figure S16. Comparison of the controlled drug release performance of the present mPEG-PCL-mPEG copolymer based delivery systems under different pH conditions, that is, pH 7.4 and pH 5. (**a**) Controlled drug release of aloin, and (**b**) controlled drug reaase of curcumin.

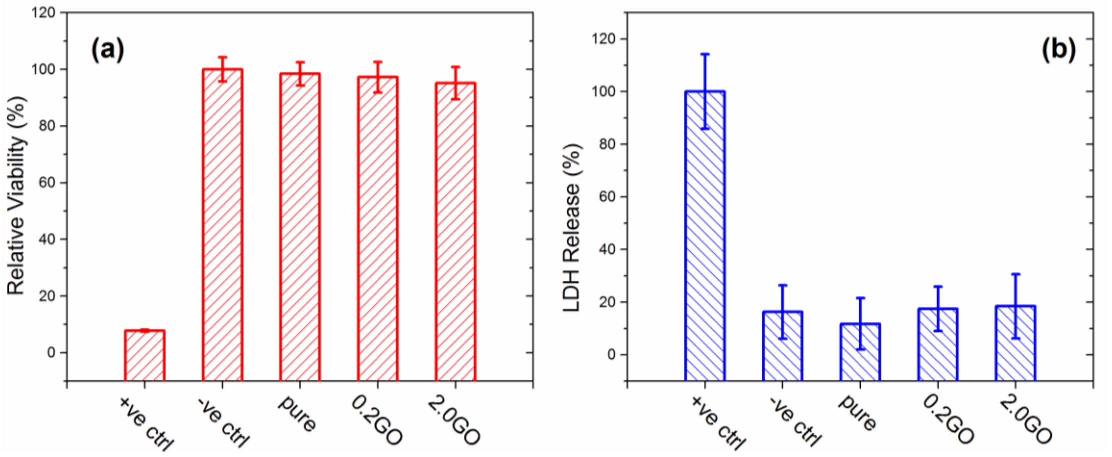

Figure S17. ***In vitro*** **cytotoxicity evaluation of pure copolymer and its nanocomposites with GO.** (**a**) The *in vitro* skin toxicity evaluation result through a cell viability measurement. (**b**) The *in vitro* skin toxicity evaluation result through a LDH release test.

Table S1. Molecular weight measurement of the synthesized copolymer mPEG-PCL-mPEG.

Copolymer mPEG-PCL-mPEG Mn [a] of specific blocks	Total Mn [a] (Theoretical)	Total Mn [b] (^{1}H-NMR)	Total Mn [c] (GPC)	PDI [d] (GPC)
550-2000-550	3100	3246	3291	1.12

Note:

[a] Theoretical value of number-average molecular weight (Mn);

[b] Calculated from ^{1}H-NMR results;

[c] Calculated from GPC results;

[d] Polydispersity (M_w /M_n) (as calculated by GPC).
